# Acute cardiac dysfunction and rapid descending aortic expansion following surgery for acute Stanford type A aortic dissection: a case report

**DOI:** 10.1093/ehjcr/ytag082

**Published:** 2026-01-31

**Authors:** Koki Yokawa, Taku Nakagawa, Yosuke Tanaka, Kazunori Yoshida, Hidetaka Wakiyama

**Affiliations:** Department of Surgery, Division of Cardiovascular Surgery, Kakogawa Central City Hospital, 439, Honmachi, Kakogawachou, Kakogawa 675-8611, Japan; Department of Surgery, Division of Cardiovascular Surgery, Kakogawa Central City Hospital, 439, Honmachi, Kakogawachou, Kakogawa 675-8611, Japan; Department of Surgery, Division of Cardiovascular Surgery, Kakogawa Central City Hospital, 439, Honmachi, Kakogawachou, Kakogawa 675-8611, Japan; Department of Surgery, Division of Cardiovascular Surgery, Kakogawa Central City Hospital, 439, Honmachi, Kakogawachou, Kakogawa 675-8611, Japan; Department of Surgery, Division of Cardiovascular Surgery, Kakogawa Central City Hospital, 439, Honmachi, Kakogawachou, Kakogawa 675-8611, Japan

**Keywords:** Frozen elephant trunk, Acquired coarctation of the aorta, Cardiac dysfunction, Left ventricular afterload, Case report

## Abstract

**Background:**

Severe true lumen stenosis of the aorta can increase left ventricular afterload and impair cardiac function. We report a rare case of significant true lumen stenosis and cardiac dysfunction after undergoing total arch replacement for acute type A aortic dissection.

**Case summary:**

A 39-year-old man with acute Stanford type A aortic dissection and intimal tear in the ascending aorta underwent the Bentall procedure and total arch replacement with a frozen elephant trunk (FET). He was readmitted 1 month after discharge with progressive cardiac dysfunction. Myocardial scintigraphy excluded ischaemia, and computed tomography revealed severe true lumen stenosis at the distal FET, resulting in an elevated left ventricular afterload. Thoracic endovascular aortic repair (EVAR) to expand the true lumen resulted in gradual improvement in cardiac function. However, there was progressive dilation of the descending and thoracoabdominal aorta: 26 mm at onset and 32 and 46 mm at 2 and 6 months postoperatively, respectively. To close distal re-entries, EVAR was performed after 2 months. His brain natriuretic peptide level decreased from 2901 to 142 pg/ml over 8 months; open abdominal aortic replacement was performed 9 months after the onset, after sufficient cardiac function recovery (ejection fraction = 40%).

**Discussion:**

True lumen stenosis is a substantial reversible cause of cardiac dysfunction after type A dissection surgery. Thoracic EVAR effectively reduces the afterload and restores cardiac function; long-term imaging and staged intervention are essential to address distal aortic remodelling.

Learning points

- Frozen elephant trunk can promote true lumen expansion in acute aortic dissection, but may fail to enlarge the distal true lumen.
- True lumen narrowing may result in increased afterload and severe cardiac dysfunction, with delayed recovery if not promptly addressed.
- Persistent false lumen perfusion can cause progressive aortic enlargement even after re-entry closure, necessitating careful long-term monitoring.

## Introduction

The frozen elephant trunk (FET) technique used in the repair of Stanford type A acute aortic dissection promotes expansion and favourable remodelling of the true lumen, extending from the descending thoracic aorta to the more distal segments.^1,2^ In some cases, persistent false lumen flow is present, and distal true lumen expansion is not observed, with some cases exhibiting true lumen narrowing. Although true lumen narrowing rarely results in cardiac dysfunction, we present a unique case where severe distal true lumen narrowing increased the left ventricular afterload, resulting in considerable cardiac dysfunction, along with rapid expansion of the descending aorta because of residual false lumen flow.

## Summary figure

**Figure:**
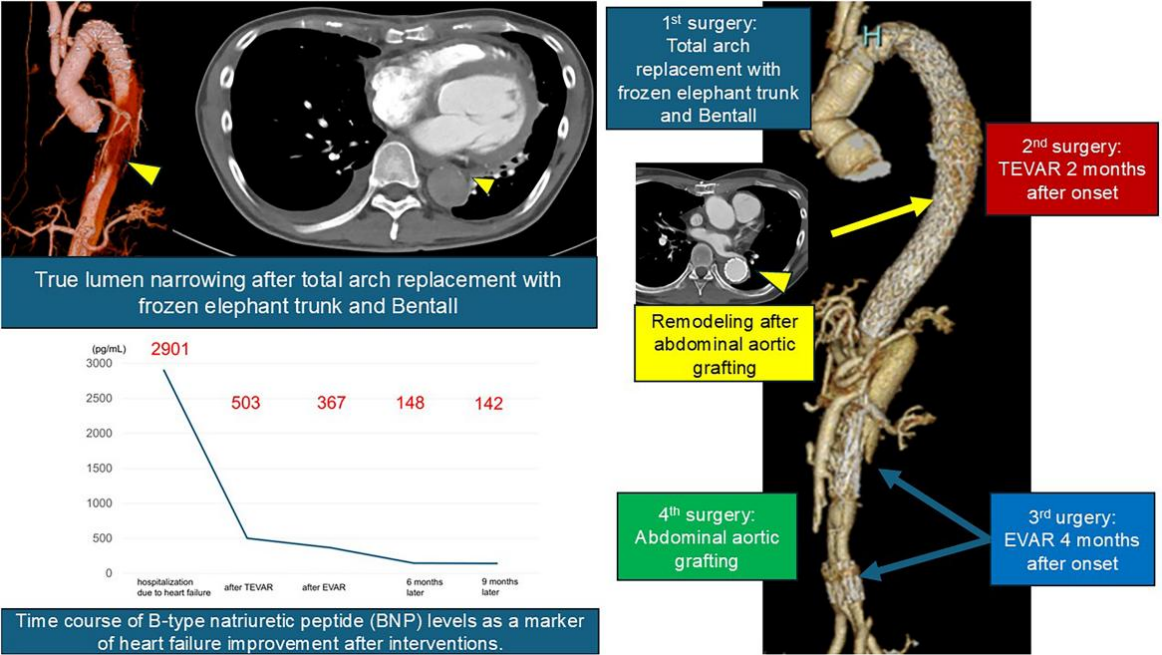


## Case report

A 39-year-old man presented with acute Stanford type A aortic dissection with an intimal tear in the ascending aorta. The patient had a medical history of bronchial asthma and atopic dermatitis, with no other notable comorbidities or chronic medication use. No physical findings suggestive of a hereditary connective tissue disorder were observed, and the aortic valve was confirmed to be tricuspid. Notably, the patient’s mother had died suddenly of acute aortic dissection. He underwent the Bentall procedure using a mechanical valve, combined with total arch replacement using a Thoraflex™ Hybrid prosthesis. During surgery, the patient developed ventricular fibrillation following weaning from cardiopulmonary bypass, requiring extracorporeal membrane oxygenation (ECMO). After intensive management, he was successfully weaned off ECMO and discharged home at 1 month postoperatively. Although contrast-enhanced computed tomography (CT) is routinely performed at our institution following the repair of acute type A aortic dissection, the patient was discharged after non-contrast CT evaluation, as the attending physician at that time deemed it sufficient for postoperative assessment.

**Figure 1:**
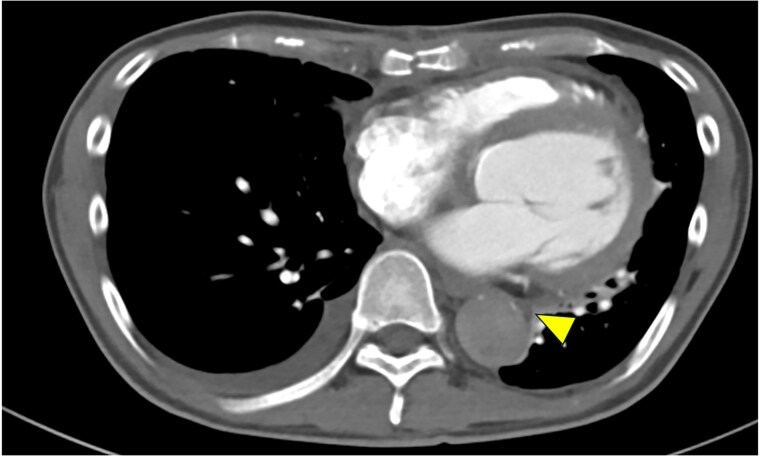
Contrast-enhanced axial computed tomography showing marked narrowing of the true lumen of the descending aorta (yellow arrowhead) due to false lumen expansion.

Two months after the initial surgery, he was readmitted due to symptoms of heart failure, before the first scheduled outpatient follow-up. Chest X-ray performed upon readmission to the emergency department demonstrated severe pulmonary congestion, consistent with acute heart failure. Laboratory tests on admission revealed an elevated B-type natriuretic peptide (BNP) level of 2901 pg/ml (normal range: <18.4 pg/ml) and a PT-INR of 2.1 (therapeutic range for mechanical valve: 2.0–3.0), indicating appropriate anticoagulation for the mechanical aortic valve. Results of liver function tests (aspartate aminotransferase, alanine aminotransferase) and renal function tests (serum creatinine) were within normal ranges. Transthoracic echocardiography revealed marked left ventricular dilation (left ventricular end-diastolic diameter/end-systolic diameter: 62/53 mm) and severely reduced systolic function, with a left ventricular ejection fraction (LVEF) of 20% in the absence of marked valvular disease. Contrast-enhanced CT showed no abnormality at the coronary reimplantation sites, and myocardial scintigraphy ruled out myocardial ischaemia. After the initial surgery, the patient experienced transient paroxysmal atrial fibrillation but was in normal sinus rhythm at the time of readmission. However, CT imaging revealed severe true lumen narrowing at the distal end of the FET (*Figure 1*). Accordingly, treatment for heart failure was initiated. Dobutamine was administered at a rate of approximately 2.6 µg/kg/min for 4 days after admission. Thereafter, guideline-directed medical therapy for heart failure was initiated, which included bisoprolol (1.5625 mg/day), losartan potassium (12.5 mg/day), spironolactone (25 mg/day), ivabradine (5 mg/day), pimobendan (2.5 mg/day), and azosemide (15 mg/day), under the supervision of the cardiology team.

The cardiac dysfunction was attributed to the increased left ventricular afterload caused by the narrowing of the true lumen. Accordingly, thoracic endovascular aortic repair (TEVAR) was performed to expand the true lumen in the descending thoracic aorta using a conformable TAG endograft at 2 months after the onset of acute aortic dissection. After TEVAR, the BNP levels decreased to 503 pg/ml; however, the LVEF showed no considerable improvement, remaining at 26%.

**Figure 2:**
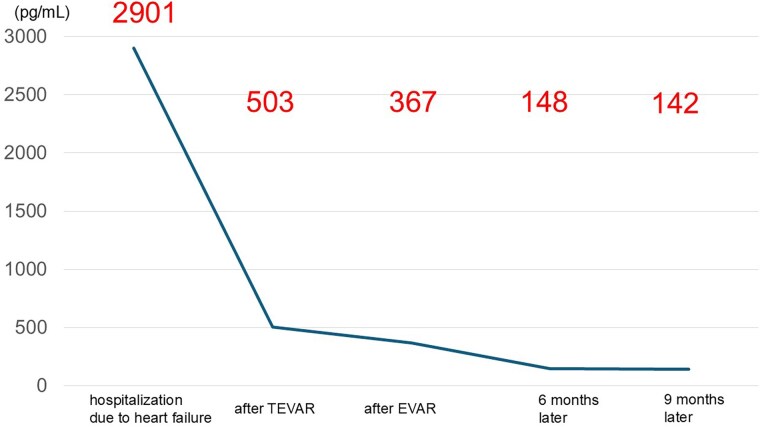
Time-course data of B-type natriuretic peptide (BNP) levels as a marker of heart failure improvement. The BNP level decreased from 2901 pg/ml at admission to 503 pg/ml after thoracic endovascular aortic repair (TEVAR), 367 pg/ml after endovascular aneurysm repair (EVAR), and further to 148 and 142 pg/ml at the 6- and 9-month follow-up assessments, respectively.

**Figure 3:**
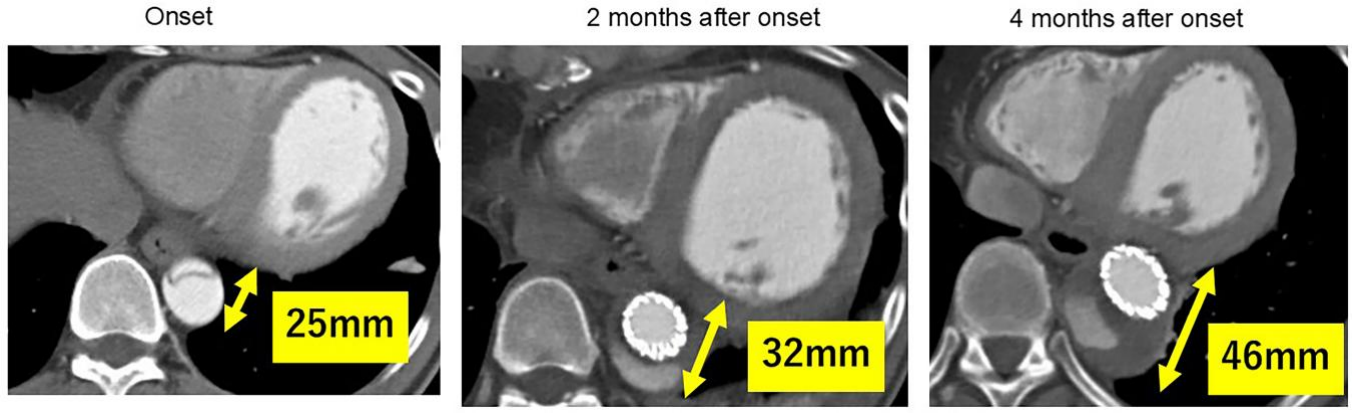
Time-course data of the aortic diameter showing progressive enlargement on serial contrast-enhanced computed tomography. The maximum diameter increased from 25 mm at onset to 32 and 46 mm after 2 and 4 months after onset, respectively, prompting surgical intervention.

Progressive enlargement of the descending aorta was observed, indicating persistent false lumen perfusion. The aortic diameter increased from 25 mm at the time of symptom onset to 32 mm at 2 months after onset (following TEVAR). Given that the dilatation continued to worsen during the outpatient follow-up, an abdominal endovascular aortic repair (EVAR) was performed to close the distal re-entry at 4 months after symptom onset. A VIABAHN^®^ stent graft was deployed in the left renal artery, and the AFX^®^ endograft system was used for the EVAR. However, despite undergoing EVAR, the descending aorta continued to enlarge, reaching 46 mm at 4 months after symptom onset. Intraoperative angiography revealed residual false lumen perfusion from the re-entry points of both the internal iliac arteries. After EVAR, the BNP levels declined consistently (*Figure 2*). At 9 months after the initiation of heart failure treatment, LVEF had improved to 40%, accompanied by a reduction in left ventricular dilation; however, the aortic diameter continued to enlarge, with a maximum diameter of the descending aorta of 50 mm). *Figure 3* shows the chronological changes in the descending aortic diameter from the time of onset.

**Figure 4:**
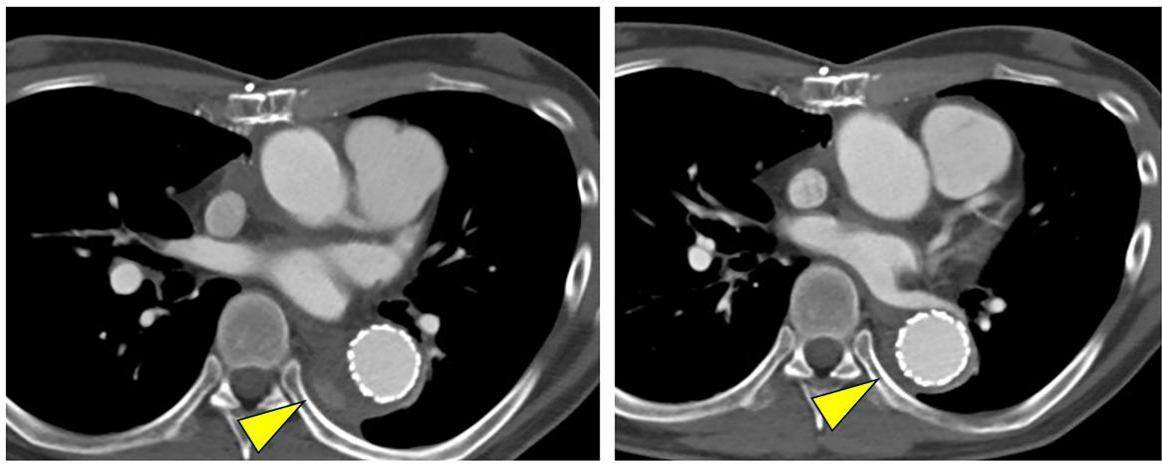
Pre- and postoperative contrast-enhanced computed tomography scans illustrating a marked reduction in the false lumen following abdominal aortic replacement.

**Figure 5:**
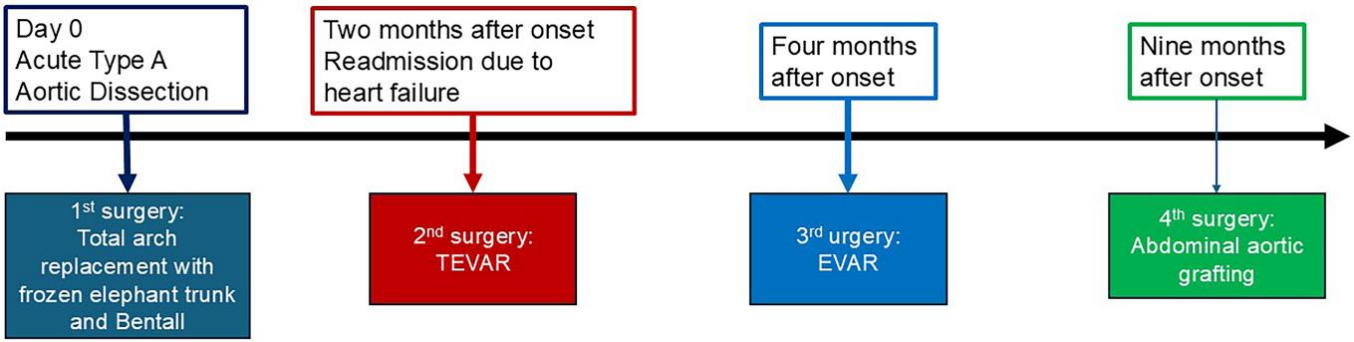
A timeline illustrating the patient’s clinical course: acute type A aortic dissection on Day 0; underwent initial total arch replacement with a frozen elephant trunk and Bentall procedure; readmission at 2 months after symptom onset followed by thoracic endovascular aortic repair (TEVAR); endovascular aneurysm repair (EVAR) at 4 months due to persistent aortic enlargement; and abdominal aortic grafting at 9 months after symptom onset.

Considering the degree of cardiac dysfunction and the extensive invasiveness of previous aortic surgeries, thoracoabdominal aortic replacement was deemed too burdensome. Therefore, an abdominal aortic replacement was performed to eliminate false lumen perfusion into the descending aorta. Postoperative contrast-enhanced CT showed that, although residual false lumen perfusion persisted in the thoracoabdominal aorta, the false lumen in the descending aorta had been successfully excluded (*Figure 4*). A timeline summarizing the chronological sequence of the patient’s surgical and clinical course is presented in *Figure 5*. Subsequently, pimobendan was discontinued. At 16 months after the onset of acute aortic dissection, the aortic diameter remained stable without further enlargement, and the patient had no recurrence of heart failure.

## Discussion

Acquired coarctation of the aorta is a rare complication of total aortic arch replacement. Anegawa *et al*.^3^ reported a case of acquired aortic coarctation after total aortic arch replacement using a conventional elephant trunk and attributed the resulting heart failure to increased afterload caused by narrowing at the elephant trunk segment. The patient showed clinical improvements after undergoing TEVAR.

Similarly, in the present case, heart failure was likely precipitated by the increased afterload due to severe true lumen narrowing at the distal end of the FET. True lumen compression or residual aortic obstruction with chronic aortic dissection can elevate the left ventricular afterload and impair diastolic function.^4^ We hypothesized that the initial decline in LVEF predominantly resulted from the increased afterload due to true lumen narrowing. Subsequent stabilization of the afterload, along with guideline-directed medical therapy, likely contributed to reverse remodelling and the partial recovery of LVEF observed at follow-up.

The PETTICOAT technique reportedly is an effective adjunct for preventing true lumen collapse and for promoting distal aortic remodelling after TEVAR.^5^ In the present case, its application at the time of TEVAR might have contributed to the true lumen stabilization and improved haemodynamic recovery.

Notably, persistent false lumen perfusion is a well-known factor associated with unfavourable aortic remodelling and subsequent aneurysmal dilatation after the surgical repair of type A dissection. Recently, incomplete thrombosis of the false lumen has been reported to increase the risk of distal aortic enlargement and secondary intervention.^6^ Additionally, anticoagulation therapy for the mechanical valve was appropriately maintained throughout the clinical course, with the INR values remaining within the therapeutic range. Previous studies have suggested that anticoagulation may increase the likelihood of false lumen patency and hinder favourable aortic remodelling after type A dissection repair.^7,8^ In our case, persistent false lumen perfusion, despite adequate surgical and endovascular interventions, may have been influenced by the patient’s anticoagulation status. In clinical practice, such enlargement of the descending aorta after total arch replacement using an FET is rarely seen. The persistent aortic enlargement observed even after TEVAR and EVAR further corroborates that the substantial false lumen perfusion via re-entry tears contributed to disease progression.

The delayed recovery of cardiac function was considered to be due to the late initiation of treatment for true lumen narrowing. Given that the patient did not present with overt symptoms of heart failure during hospitalization, performing early interventions was not possible. However, we acknowledge that earlier intervention would have led to a more rapid improvement in cardiac function. Furthermore, the present case highlights the importance of early and serial postoperative imaging surveillance after surgery for acute type A dissection. In the present case, true lumen narrowing was detected only after the patient presented with heart failure symptoms, as no contrast-enhanced CT was performed before readmission. Earlier postoperative CT imaging could have facilitated timely recognition and enabled early intervention, potentially preventing the development of cardiac dysfunction.

## Conclusion

Residual true lumen narrowing can persist even after total arch replacement using the frozen elephant trunk technique for acute type A aortic dissection and may result in clinically significant cardiac dysfunction due to increased left ventricular afterload. This case underscores the importance of careful and serial postoperative imaging surveillance to enable early detection and timely intervention for graft-related anatomical complications.

## Lead author biography


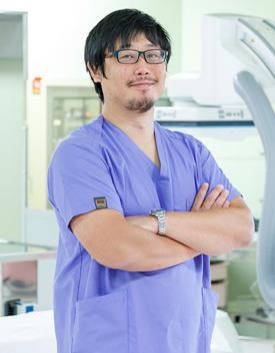


Dr. Koki Yokawa is a staff surgeon in the Department of Cardiovascular Surgery at Kakogawa Central City Hospital, Kakogawa, Japan.

**Consent:** The authors confirm that written consent for submission and publication of this case report including images and associated text has been obtained from the patient, in line with COPE guidance.

## Data Availability

The data pertaining to this article can be shared upon reasonable request to the corresponding author.
